# Hepcidin deficiency in mice impairs white adipose tissue browning possibly due to a defect in de novo adipogenesis

**DOI:** 10.1038/s41598-023-39305-0

**Published:** 2023-08-07

**Authors:** Jean-Christophe Deschemin, Céline Ransy, Frédéric Bouillaud, Soonkyu Chung, Bruno Galy, Carole Peyssonnaux, Sophie Vaulont

**Affiliations:** 1 Institut Cochin, INSERM, CNRS, Université Paris Cité, 75014 Paris, France; 2 Laboratory of Excellence GR-Ex, Paris, France; 3 Department of Nutrition, University of Massachusetts-Amherst, Amherst, MA 01003 USA; 4 German Cancer Research Center, “Division of Virus-Associated Carcinogenesis”, Im Neuenheimer Feld 280, 69120 Heidelberg, Germany

**Keywords:** Physiology, Endocrinology

## Abstract

The role of iron in the two major sites of adaptive thermogenesis, namely the beige inguinal (iWAT) and brown adipose tissues (BAT) has not been fully understood yet. Body iron levels and distribution is controlled by the iron regulatory peptide hepcidin. Here, we explored iron homeostasis and thermogenic activity in brown and beige fat in wild-type and iron loaded Hepcidin KO mice. Hepcidin-deficient mice displayed iron overload in both iWAT and BAT, and preferential accumulation of ferritin in stromal cells compared to mature adipocytes. In contrast to BAT, the iWAT of Hepcidin KO animals featured with defective thermogenesis evidenced by an altered beige signature, including reduced UCP1 levels and decreased mitochondrial respiration. This thermogenic modification appeared cell autonomous and persisted after a 48 h-cold challenge, a potent trigger of thermogenesis, suggesting compromised de novo adipogenesis. Given that WAT browning occurs in both mice and humans, our results provide physiological results to interrogate the thermogenic capacity of patients with iron overload disorders.

## Introduction

Iron is an essential element for a wide variety of fundamental biological processes (cellular respiration, DNA synthesis, proliferation…); yet iron overload can be deleterious by triggering free radicals and oxidative stress. Consequently, iron concentrations in cells and tissues must be controlled by mechanisms that complement and fine-tune systemic regulation of iron metabolism^1^.

The importance of iron metabolism in the biology of the adipose tissue (AT) is being increasingly recognised^2^. Indeed, AT plays a key role in the regulation of systemic energy levels, while iron, as a component of vital enzymes for ATP synthesis in mitochondria, is intimately linked to energy metabolism^3^.

Two general classes of adipocytes are present in mammals: white and brown. While white adipocytes store and release energy as fatty acids in response to systemic demand, brown adipocytes burn substrates to produce heat, a process known as thermogenesis. The thermogenic activity of brown fat cells relies on Uncoupling Protein 1 (UCP1). UCP1 is localised on the inner membrane of mitochondria and uncouples oxidative respiration from ATP synthesis, resulting in the dissipation of energy derived from substrate oxidation as heat^4^.

Importantly, UCP1-expressing adipocytes also develop within white adipose tissue (WAT) in response to thermogenic stimuli (cold temperature and β3-adrenergic receptor agonists), these fat cells being termed ‘beige’ or ‘brite’ adipocytes. Consistent with their ability to execute thermogenesis, brown and beige adipocytes share morphological features. In comparison to white adipocytes, they display small multilocular lipid droplets, are rich in mitochondria, and can activate the thermogenic gene program^5^. Brown and beige adipocytes are however considered distinct cells based on their different developmental origins and functional characteristics^6^.

Brown adipocytes arise during embryogenesis from myogenic precursor cells and constitutively express high levels of proteins of the thermogenic machinery. Beige adipocytes develop within the WAT during the postnatal period in response to beiging signals. Recruitment of beige adipocytes may arise through de novo differentiation from resident precursor cells (‘de novo biogenesis’) or through white-to-beige conversion (transdifferentiation) of mature white adipocytes^6^. The relative contribution of each pathway varies with the thermogenic stimulus^7^.

At the whole body level, iron homeostasis is mediated by the iron-regulatory hormone hepcidin (encoded by *Hamp1*). Hepcidin is primarily produced in hepatocytes and controls plasma and tissue iron levels by regulating the delivery of iron to plasma via the iron export protein ferroportin (*Slc40a1*, Solute Carrier Family 40 Member 1). By interacting with ferroportin, hepcidin either occludes its efflux pathway, or triggers its lysosomal degradation in presence of iron^8,9^, resulting in intracellular iron accumulation.

At the cellular level, iron homeostasis is orchestrated post-transcriptionally by the Iron Regulatory Proteins (IRP)-1 and -2 (also known as ACO1 and IREB2, respectively). Both proteins bind to cis-regulatory RNA stem-loop structures called Iron Responsive Elements (IREs), which are present in the untranslated region (UTR) of mRNAs encoding proteins involved in iron uptake (DMT1, divalent metal transporter 1, encoded by *Slc11a2*, and TFR1, transferrin receptor 1, also known as TFRC), iron storage (ferritin H and L chains) and export (ferroportin)^1,10^. IRP binding to IRE motifs located in the 3′-UTR of the *Tfr1* and *Dmt1* transcripts leads to mRNA stabilisation. On the opposite, IRPs impose a steric constraint on mRNA translation when bound to IRE motifs present in the 5′-UTR (*ferritin*, *ferroportin*). The IRE-binding activity of both IRPs is regulated by intracellular iron levels; in iron replete cells, IRP2 is displaced from IRE RNA by F-box and leucine-rich repeat protein 5 (FBXL5) and targeted for degradation^11^, whereas IRP1 assembles an iron-sulfur cluster and functions as an aconitase^12^.

Recent evidence suggests that fine tuning of iron availability is critical for optimal AT physiology, in particular adipocyte differentiation, adipocyte protection from oxidative stress and adipocyte lipid handling^2,13^, as well as for the control of AT inflammation^14^ and AT thermoregulation^15–18^. In addition, adipocyte iron has been shown to regulate two critical adipokines, leptin^19^ and adiponectin^20^, linking iron to insulin sensitivity and food intake, respectively. Moreover, in vivo studies have reported adipose tissue dysfunction linked to key proteins of iron metabolism including TFR1^15^, ferritin^21^, heme oxygenase^22^, mitoneet^23^ and maptriptase2^24^**.**

Based on our recent observation that liver hepcidin downregulation is a fundamental mechanism required to activate thermogenesis^16^, we set out to investigate the physiology of the AT in Hepcidin KO mice (Hepc KO), a mouse model of iron overload^25^, focusing on brown and beige adipocytes that are both competent for thermogenesis.

## Results and discussion

### Expression of iron metabolism genes in adipose tissue of WT and Hepc KO mice

Iron homeostasis was examined in the AT of WT and Hepc KO animals. Experiments were conducted using the interscapular region as brown AT (BAT); as beige AT, we used the inguinal WAT (iWAT), where beige adipocytes are the most abundant.

**Figure 1:**
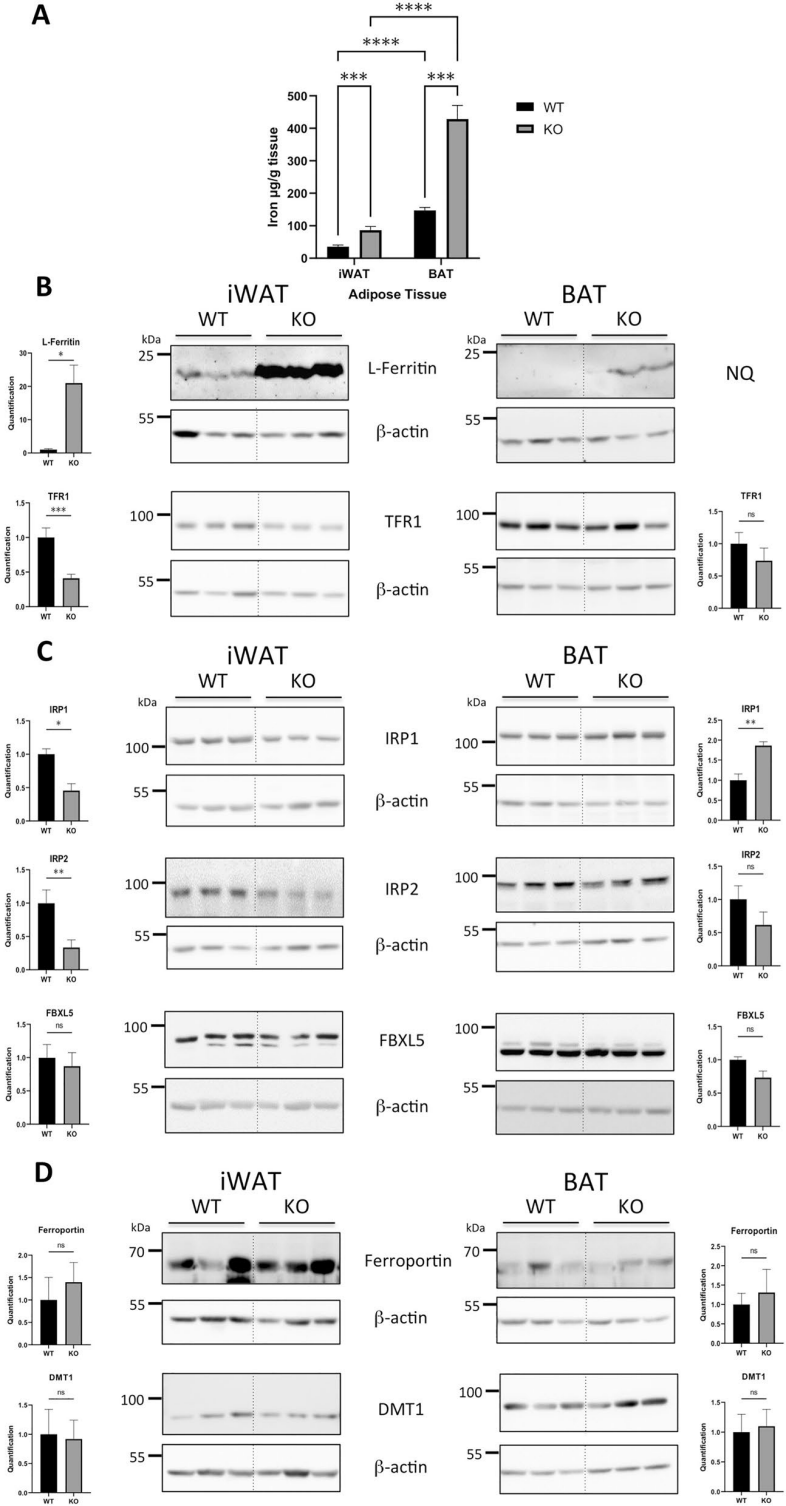
Iron content and expression of iron metabolism genes in iWAT and BAT of WT and Hepc KO mice: (**A**) Iron levels (µg/g wet tissue) in iWAT and BAT. Error bars represent SEM (WT, n = 7; KO, n = 4 mice). Statistical significance, assessed by an 2 way ANOVA test, is indicated by * symbols (**p < 0.01, ***p < 0.001, ****p < 0.001). (**B**–**D**) WB analysis of l-Ferritin (**B**, top), TFR1 (**B**, bottom), IRP1 (**C**, top), IRP2 (**C**, middle), FBXL5 (**C**, bottom), FPN (**D**, top) and DMT1 (**D**, bottom) protein expression in iWAT (left panels) and BAT (right panels). l-Ferritin levels were analysed using cytosolic extracts. All other proteins were analysed using whole tissue extracts. Protein expression was normalised to β-actin and quantified using Image J. For each AT tissue, quantification of the blots is presented relative to WT. The dotted lines between WT and KO animals in the WB are to denote the separation of the groups (the blots were not modified). Similar results were obtained in at least one independent experiment. Error bars represent SEM for n = 3 mice in each group. Statistical significance is indicated by * symbols (*p < 0.05, **p < 0.01, ***p < 0.001). Similar results were obtained in at least one independent experiment. NS is for non significant.

In agreement with the high mitochondrial content of the BAT, WT mice had six times more iron in the BAT than in iWAT (Fig. 1A). Compared to WT, KO mice displayed a threefold increase in total iron levels, in both iWAT and BAT (Fig. 1A). This represents a relatively modest accumulation of iron compared to the more than 20-fold increase in iron levels in the liver and pancreatic parenchyma of these mice^25^.

At the molecular level, the iWAT of Hepc KO mice exhibited the typical signature of iron-accumulating tissues, with a massive increase in l-ferritin protein expression, a marker of iron accumulation, and suppression of TFR1 (Fig. 1B, left). These alterations in ferritin and TFR1 expression could be, at least in part, accounted for by a decrease in the protein levels of IRP1 and/or IRP2 (Fig. 1C). The down regulation of IRP1 was accompanied by a mild reduction in the expression of the corresponding mRNA (Supplementary Fig. 1, top left panel). By contrast, *Irp2* transcript levels remained unchanged (Supplementary Fig. 1, top right panel). Since IRP2 is principally regulated through E3 ubiquitin ligase FBXL5-mediated degradation^11^, we anticipated that an increase in FBXL5 in the iWAT of Hepc KO mice would explain the decrease in IRP2. However, FBXL5 protein levels in iWAT were similar between WT and Hepc KO mice (Fig. 1C).

Although iron levels in the BAT of WT mice were 6 times higher than in iWAT, ferritin expression remained below the detection level (Fig. 1B, right). As reported previously^18^, TFR1 protein expression was elevated in BAT when compared to iWAT (Fig. 1B, bottom). Surprisingly and in contrast to iWAT, TFR1 expression in BAT was not decreased in Hepc KO animals (Fig. 1B). Similarly, hepcidin deficiency did not alter IRP2 and FBXL5 levels in BAT, and even enhanced IRP1 expression (Fig. 1C and Supplementary Fig. 1).

Since hepcidin can induce ferroportin degradation, we expected an accumulation of the exporter in the AT of hepcidin deficient animals. However, ferroportin protein levels remained unchanged in iWAT and BAT of Hepc KO mice (Fig. 1D). Recent structural studies revealed that hepcidin preferentially interacts with and regulates ferroportin molecules that actively transport iron^9^. It is plausible that hepcidin spares ferroportin molecules in adipocytes due to relatively low iron export activity. Supporting this notion, Britton et al. reported that adipocyte-specific ferroportin deletion in mice does not induce adipocyte iron loading^26^. Overall, the hepcidin-ferroportin axis may not be a major contributor to iron homeostasis in adipocytes, at least under steady-state conditions. Similar to ferroportin, DMT1 expression in iWAT and BAT was not affected by hepcidin deficiency (Fig. 1D).

Collectively, these results reveal marked differences in iron management between iWAT and BAT. Despite a significantly higher iron content, ferritin expression in BAT remains substantially lower than in iWAT. Furthermore, TFR1 is not suppressed upon iron loading in Hepc KO mice. Among other possibilities, the high amount of iron in BAT cells may not be sensed as excess because most of the metal is engaged in heme and iron-sulfur cluster prosthetic groups to sustain the high mitochondrial iron demand of brown adipocytes.

### Cellular iron loading pattern in the AT of Hepc KO mice

**Figure 2:**
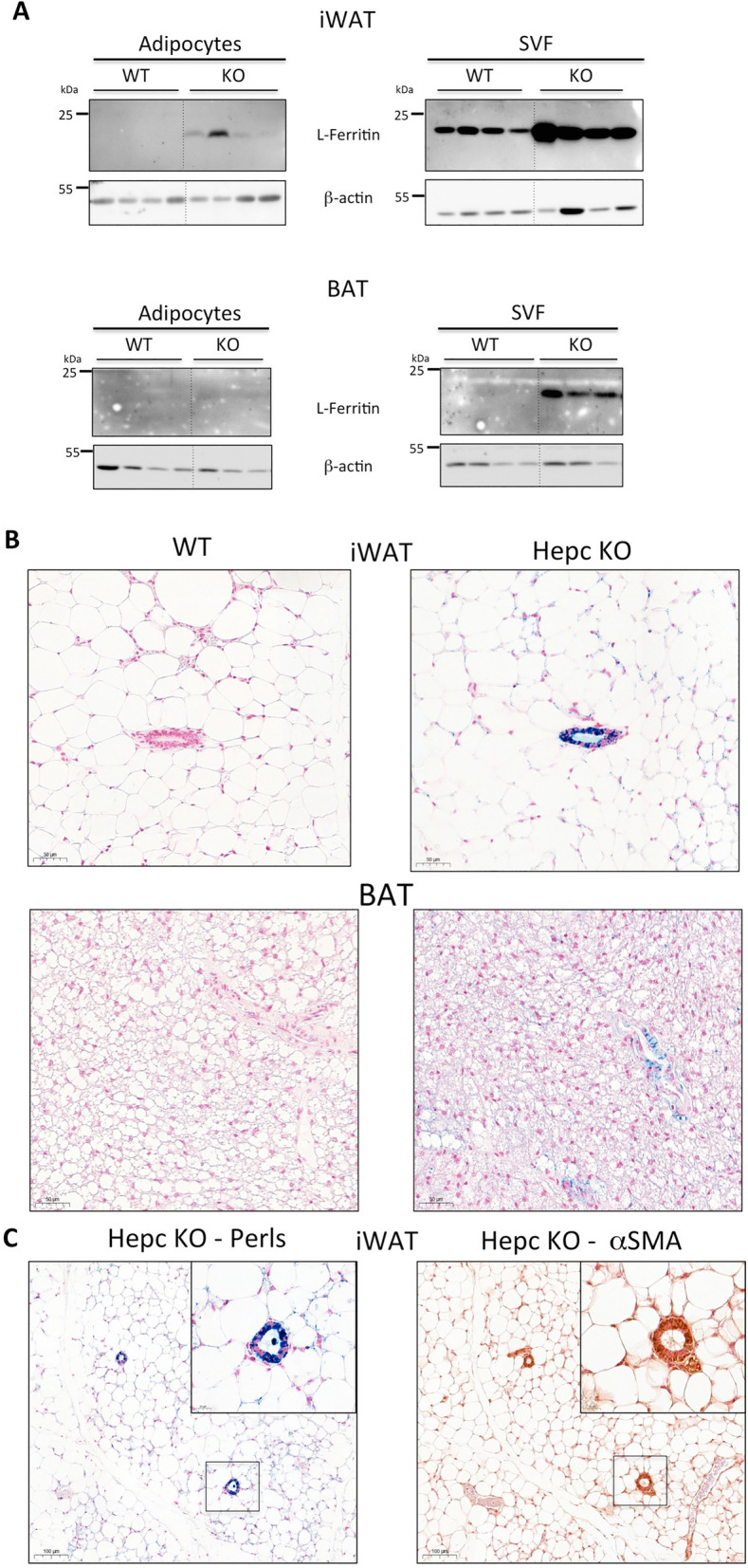
Ferritin and iron distribution in AT of WT and Hepc KO mice. (**A**) WB analysis of l-Ferritin in adipocyte (left panels) versus stromal fractions (right panels) from the iWAT (top) and BAT (bottom) of WT and Hepc KO mice. The dotted lines between WT and KO animals in the WB are to denote the separation of the groups (the blots were not modified). (**B**) Iron deposition in iWAT and BAT sections of WT and Hepc KO animals was visualised by Perls’ blue staining. (**C**) Adjacent sections from the iWAT of Hepc KO mice were stained with Perls’ blue (left) or with an antibody against αSMA (right). The magnification inserts show co-localisation of iron deposits with in cells of the vascular wall. In (**B**) and (**C**), nuclear fast red was used as counterstain.

Although adipocytes are the primary parenchymal cell type in AT, they are embedded in a rich stromal microenvironment composed of various cell types including immune, neuronal, and vascular cells, as well as adipose stem and progenitors cells. We therefore analysed iron metabolism parameters in the adipocyte versus stromal fraction of AT. In the iWAT, l-ferritin, was predominant in the stromal fraction, both in WT and Hepc KO mice. In adipocytes, l-ferritin was detected only in the iWAT of KO animals (Fig. 2A, top).

In the BAT, l-ferritin was detected exclusively in the stromal fraction of Hepc KO mice (Fig. 2A, bottom).

To determine which cell type accumulates iron in the AT, adipose sections were stained with Perl's Prussian blue (Fig. 2B). In WT mice, iWAT and BAT presented with no detectable staining. In contrast, specific iron staining was observed in the iWAT of Hepc KO mice, with faint iron deposits in adipocytes and some stromal cells and marked accumulation of the metal in blood vessel walls, most likely in vascular smooth muscle cells as judged by co-localisation of the iron signal with alpha-smooth muscle actin (α-SMA) (Fig. 2C). A similar, although weaker, iron staining pattern was observed in the BAT of Hepc KO animals.

Altogether, these data indicate that iron is mainly present in the stromal fractions of the iWAT in both WT and KO mice. It would be particularly interesting to further address which cells are specifically iron loaded and how this could affect the overall physiology of the AT.

### Hepcidin deficiency alters the beige signature in the iWAT

To determine whether iron accumulation in the iWAT of Hepc KO mice alters the beige signature of this tissue, we first examined UCP1, which stimulates thermogenesis by uncoupling cellular respiration from mitochondrial ATP synthesis to produce heat.

**Figure 3:**
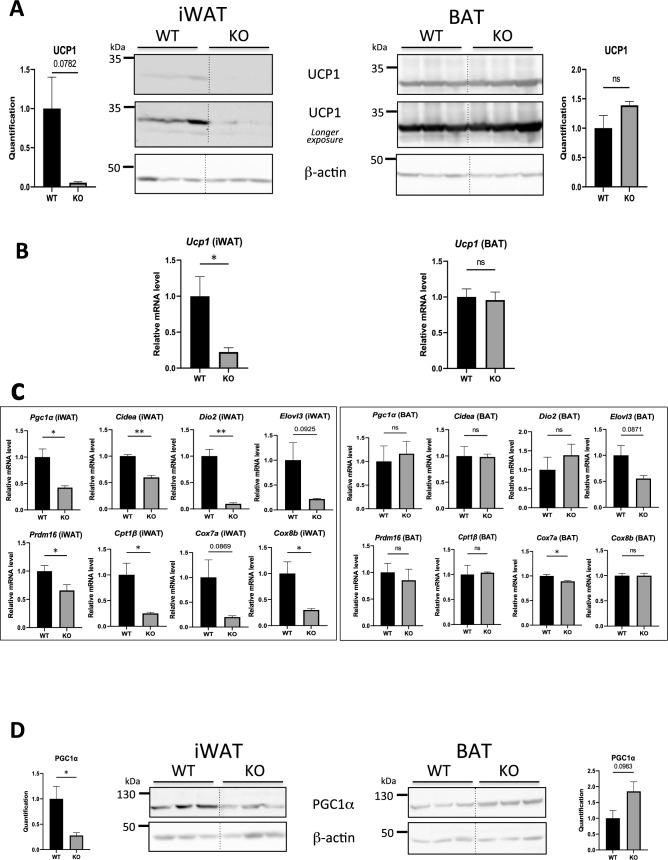
Beigeing-related gene expression in ATs of WT versus Hepc KO mice: (**A**) WB analysis of UCP1 levels in total extracts from iWAT and BAT of WT and Hepc KO mice. The dotted lines between WT and KO animals denote the separation of the groups (the blots were not modified). (**B**,**C**) Real time PCR analysis of *Ucp1* mRNA levels and beige markers in iWAT and BAT, relative to *Cyclophilin-a*/*Ppia* mRNA. For each marker and AT type, the histograms display changes in expression relative to WT mice. (**D**) WB analysis of PGC1α levels in total protein extracts. In (**B**) and (**D**), expression was normalised to β-actin and quantified using Image J. Quantification of the blots is presented relative to WT in each AT. The dotted lines between WT and KO animals in the WB denote a gap in the lanes of the images of a same blot cropped together for simplicity. Error bars represent SEM for n = 3 mice in each group. Statistical significance is indicated by * symbols (*p < 0.05, **p < 0.01). Similar results were obtained in at least two independent experiments.

In BAT, basal expression of UCP1 was constitutively high, and UCP1 mRNA and protein levels were not significantly affected by hepcidin deficiency (Fig. 3A,B, right panels). In iWAT, UCP1 expression was much lower than in BAT and, importantly, its expression was markedly reduced in Hepc KO mice, at the mRNA level (Fig. 3B), as well as at the protein level (although not significantly due to the high variability of UCP1 expression in WT mice, Fig. 3A).

We next analysed a panel of beige-selective and mitochondria-related genes (Fig. 3C). Similar to *Ucp1*, all transcripts were drastically suppressed (*Pgc1α*, *Cidea*, *Dio2*, *Elovl3*, *Prdm16*, *Cpt1b*, *Cox7a1*, *Cox8b*) in the iWAT of Hepc KO mice compared to WT. Further supporting a reduction of the beige signature, the iWAT of Hepc KO mice displayed a significant decrease in the protein level of PGC1α, (peroxisome proliferator-activated receptor-γ coactivator) (Fig. 3D), a master regulator of UCP1 and activator of adaptive thermogenesis^5^. In contrast, the BAT of Hepc KO animals exhibited normal thermogenic gene expression and normal to slighgtly increased PGC1α levels (Fig. 3C,D).

These results reveal that hepcidin is required to maintain normal beige signature in the iWAT but not in the BAT. The impact of hepcidin deficiency on iWAT thermogenic gene expression does not seem to significantly affect body weight, and Hepc KO mice appeared morphologically normal. Among other possibilities, the misregulation of thermogenic genes in Hepc KO mice may not suffice to trigger changes in fat mass and/or may be antagonised by yet unknown compensatory mechanisms.

To assess whether iron could directly down-regulate PGC1α and UCP1, iWAT adipocytes from WT mice were differentiated ex vivo and cultured in the presence of iron (Fe-NTA) for 18h. As expected, iron loading of mature adipocytes resulted in a strong decrease in *Tfr1* mRNA levels. However, iron had no effect on *Pgc1α* and *Ucp1* mRNA expression (Supplemental Fig. 2), a result in support of previous data^15,18^.

We next asked whether the changes in the beigeing signature in the iWAT of Hepc KO mice could be due to low-grade inflammation. Indeed, recent reports suggest that inflammatory factors may negatively affect browning of iWAT. In particular, lipopolysaccharide (LPS) and pro-inflammatory cytokines such as IL1β (interleukin 1 β) and TNFα (tumor necrosis factor α) were shown to reduce *Ucp1* levels in adipocytes^27–29^. We thus examined the concentration of cytokine in plasma, as well as the mRNA levels of pro-inflammatory cytokines in iWAT. Hepc KO and WT animals showed comparable cytokine profiles (Supplemental Fig. 3), suggesting that inflammation is not the cause of abnormal beigeing in the iWAT of Hepc KO mice.

### Lack of hepcidin impairs mitochondrial respiration in iWAT adipocytes

**Figure 4:**
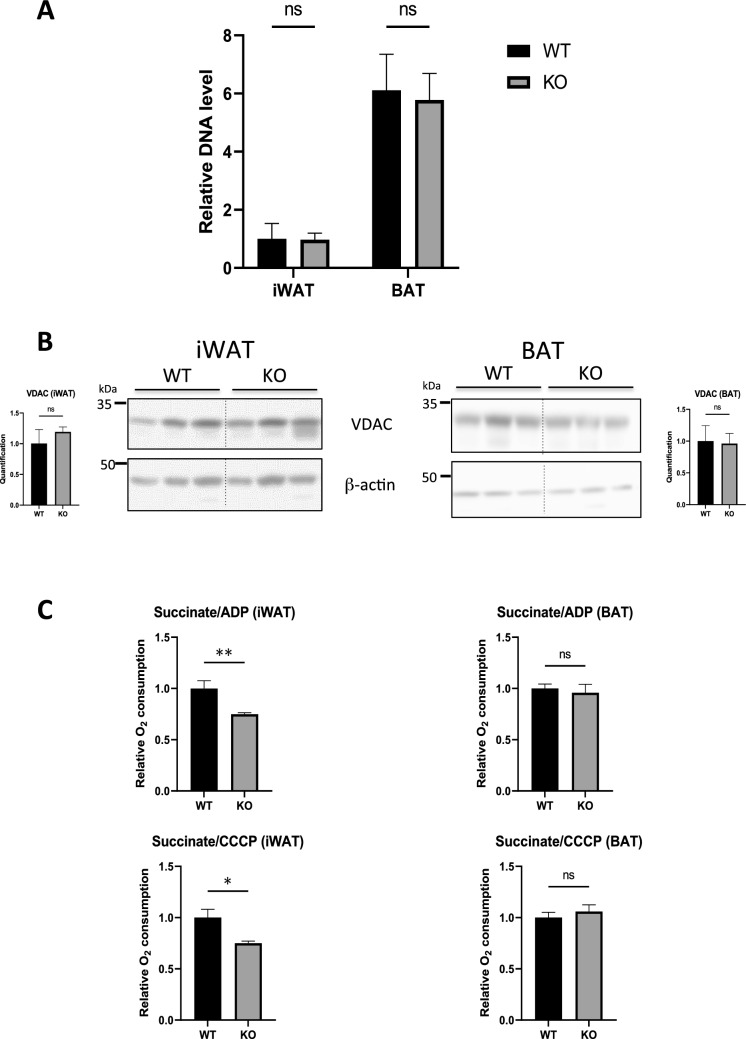
Mitochondrial function in iWAT and BAT of WT and Hepc KO mice: (**A**) The mitochondrial DNA content in iWAT and BAT was determined by real time PCR analysis of mitochondrial DNA levels after calibration to nuclear DNA. (**B**) WB analysis of VDAC levels in total protein extracts from ATs of WT versus Hepc KO mice. Expression was normalised to β-actin and quantified using Image J. The dotted lines between WT and KO animals denote the separation of the groups (the blots were not modified). Quantification of the blots is presented relative to WT mice. Error bars represent SEM for n = 3 mice in each group. (**C**) Respiration rate (mean ± SEM) in iWAT and BAT, as measured by oxygraphy (n = 5 mice per group). Statistical significance is indicated by * symbols (*p < 0.05, **p < 0.01).

Cellular iron levels are known to influence mitochondrial biogenesis^16,30^. We therefore assessed how hepcidin deficiency and iron overload affect the mitochondrial content of AT. We analysed the level of mitochondrial DNA relative to nuclear DNA using real-time PCR. As expected, the mitochondrial DNA content of the BAT was 6 times higher than that of iWAT. However, hepcidin deficiency had no impact on mitochondrial DNA levels in either iWAT or BAT (Fig. 4A). Similarly, Hepc KO mice showed no change in the expression of the mitochondrial protein VDAC compared to WT (Fig. 4B). Together, these results suggest that the abnormal browning in the iWAT of Hepc KO mice is not related to a defect in mitogenesis.

We then measured cellular oxygen consumption to determine the physiological impact of UCP1 down regulation in the AT of Hepc KO mice. For this, adipocytes were isolated from iWAT and BAT, and oxygen consumption was measured ex vivo using Oxygraph-2K for High-Resolution Respirometry with succinate as substrate. ADP is the physiological stimulator of oxygen consumption in coupled mitochondria while CCCP, carbonyl cyanide m-chlorophenylhydrazone, is a chemical protonophore that mimicked the effect of active UCP1, its optimal concentration resulting in the maximal possible rate of mitochondrial oxygen consumption.

The data are presented as the ratio of spontaneous to stimulated respiration with ADP (Fig. 4C, top) and to the maximal rate in presence of CCCP (Fig. 4C, bottom). Homogenate from BAT adipocytes from WT or Hepc KO animals showed ratios close to one hence neither ADP nor CCCP could stimulate further respiration that was already at its maximal rate in presence of substrate alone (fully uncoupled mitochondria). In contrast, with iWAT there was a significant difference between homogenate from WT (ratio≈1, fully uncoupled mitochondria) and from Hepc KO mice that showed values lower than one revealing a stimulation by ADP or CCCP hence partial coupling of mitochondria. This effect is most likely due to the down-regulation of UCP1, whose abundance may not suffice to trigger full uncoupling of mitochondrial respiration.

So far, our results suggest that hepcidin is required to maintain normal mitochondrial function and basal browning capacity in the iWAT of mice housed at room temperature, whereas it seems dispensable in BAT.

### Effect of thermogenic challenges in Hepc KO mice

**Figure 5:**
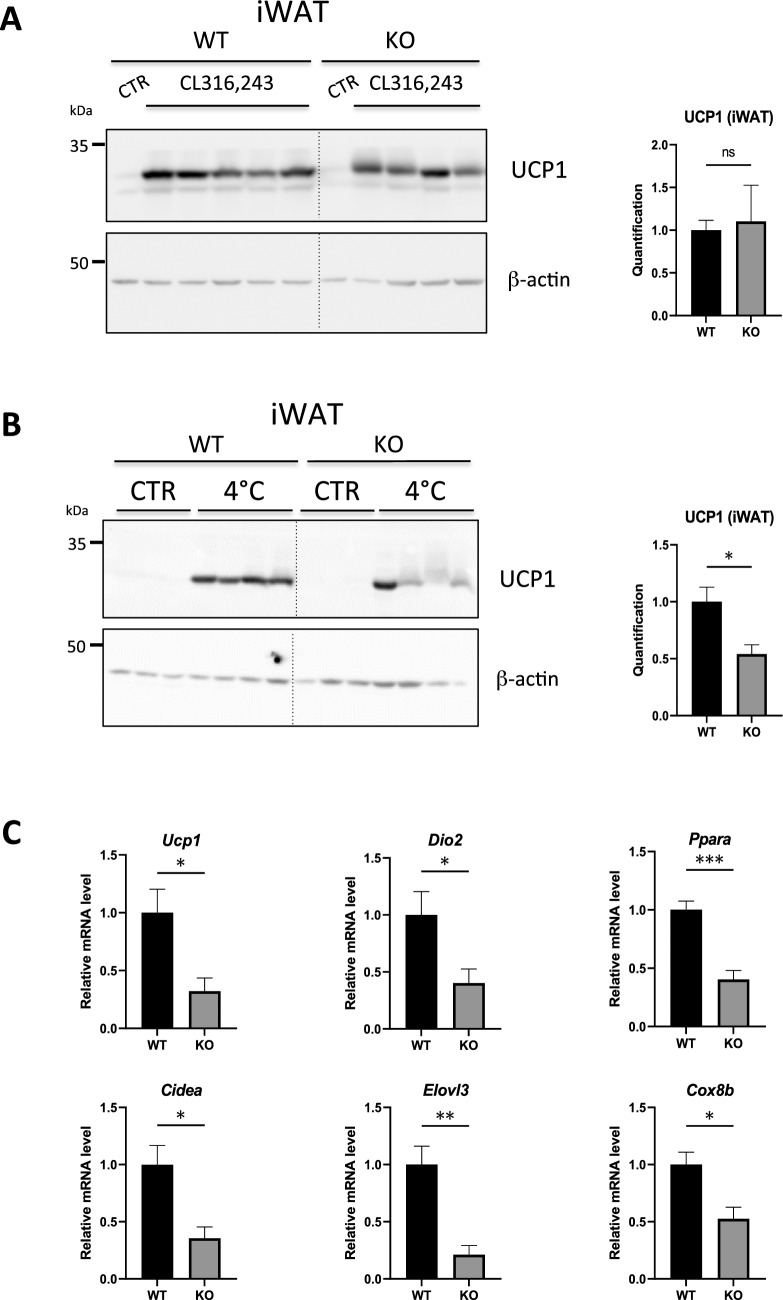
Thermogenic gene expression in iWAT of WT versus Hepc KO mice subjected to ADRB3 stimulation or to cold. (**A**) WB analysis of UCP1 levels in total protein extract from the iWAT of WT and Hepc KO mice treated with the beta-adrenergic agonist CL-316243. Mice receiving vehicle (CTR) were used as reference. (**B**) Same analysis as in (A) in mice subjected to cold stimulation (CTR: mice housed at normal temperature). Similar results were obtained in at least one independent experiment. In (**A**) and (**B**), UCP1 expression was normalised to β-actin and quantified using Image J. Quantification of the blots is presented relative to WT in each AT. The dotted lines between WT and KO animals denote the separation of the groups (the blots were not modified). (**C**) Real-time PCR analysis of *Ucp1* mRNA and beige markers relative to *Cyclophilin-a*/*Ppia* in AT from WT and Hepc KO mice after cold stimulation. Error bars represent SEM (WT, n = 5; KO, n = 4 mice) (**A**) and (WT and KO, n = 4 mice) (**B**,**C**). Statistical significance is indicated by * symbols (*p < 0.05, **p < 0.01, ***p < 0.001). Similar results were obtained in at least one independent experiment.

As iWAT is prone to induce thermogenic gene programming (‘‘beigeing/browning’’), we examined the response of WT versus Hepc KO mice to two potent thermogenic stimuli, namely cold exposure (4 °C for 48 h) and treatment with the b3-adrenergic receptor (ADRB3) agonist CL316,243 (CL). As expected, both stimuli caused potent upregulation of UCP1 in the iWAT of WT mice (Fig. 5A,B). KO mice displayed the same upregulation of UCP1 in response to β adrenergic stimulation (Fig. 5A). In contrast, UCP1 induction by cold exposure was significantly blunted in Hepc KO animals compared to WT (Fig. 5B), associated with an overall weaker induction of transcripts involved in beigeing (Fig. 5C). This data demonstrates that hepcidin is required to achieve full stimulation of thermogenic programming in the iWAT during cold exposure. Further investigation of gas exchange, whole-body energy expenditure, or physical activity with e.g. metabolic cages could help to better evaluate the metabolic and physiological consequences of abnormal thermogenic programing in Hepc KO mice.

Although cold temperature and CL have been used interchangeably to trigger beigeing, these two stimuli act through distinct mechanisms^31,32^. In particular, Jiang et al. demonstrated that unlike cold, beta-adrenergic stimulation triggers the transdifferentiation of white adipocytes into beige adipocytes through a process called ‘beige adipocyte renaissance’ (or conversion)^33^. Because hepcidin deficiency did not diminish the capacity of CL treatment to induce UCP1 and beige programming in the iWAT, it is unlikely that “beige adipocyte renaissance” is impaired in Hepc KO mice. Instead, we hypothesise a defect in de novo beige adipogenesis, i.e. in the formation of adipocytes from stem cells, which is predominantly used during acute cold stimulation^34^.

### Hepcidin deficiency does not alter beige adipocytes formation in the iWAT of juvenile mice

**Figure 6:**
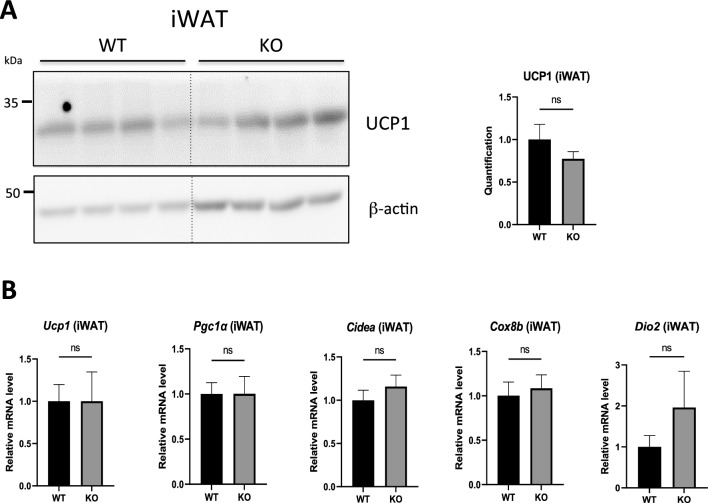
Thermogenic gene expression in iWAT during the peri-weaning period in WT and Hepc KO mice. (**A**) WB analysis of UCP1 levels in total protein extracts from the iWAT of WT versus Hepc KO mice at post-natal day 22. Expression was normalised to β-actin and quantified using Image J. Quantification of the blots is presented relative to WT. The dotted lines between WT and KO animals in the WB denote a gap in the lanes of the images of a same blot cropped together for simplicity. (**B**) Real-time PCR analysis of *Ucp1* mRNA and beige marker transcripts relative to *Cyclophilin-a*/*Ppia* in iWAT of WT and Hepc KO mice. In (**A**) and (**B**), error bars represent SEM for n = 4 mice in each group. ns is for not significant.

To further define which route of beige recruitment could be affected in the iWAT of Hepc KO mice, we analysed the browning signature at postnatal day 22, when first physiologic recruitment of beige fat occurs. During the first month of postnatal life, white AT depots undergo transient remodeling characterised by a dramatic increase in UCP1 transcript and protein expression, which peaks at 3 weeks of age^35^. This remodeling is thought to be due to transdifferentiation of preexisting adipocytes, and does not require massive proliferation and subsequent apoptosis. The surge of white fat browning is specific to white fat depots, UCP1 expression remaining unchanged in the BAT during this period. As shown in Fig. 6, WT and Hepc KO mice displayed the same browning phenotype in iWAT, with similar amounts of UCP1 protein (Fig. 6A) and browning transcripts (Fig. 6B), suggesting that beige formation during the peri-weaning period in Hepc KO mice occurs normally.

This result supports the notion that transdifferentiation of iWAT adipocytes is not affected by hepcidin deficiency.

### Cell autonomous defect in the thermogenic program of Hepc KO iWAT

**Figure 7:**
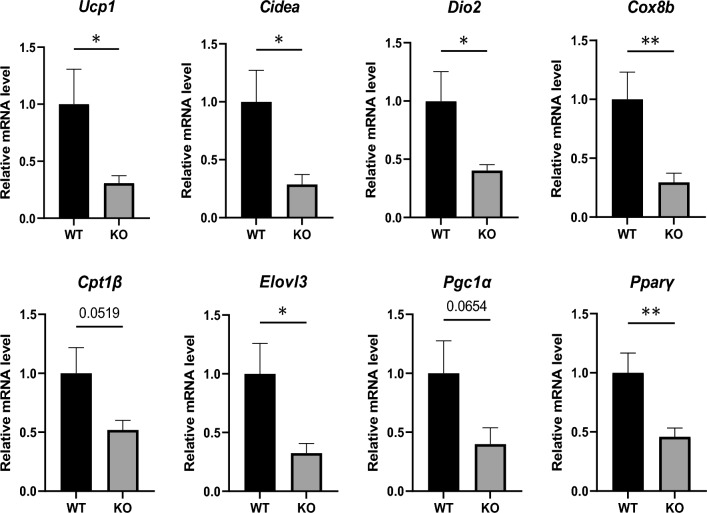
Thermogenic program in WT versus Hepc KO iWAT adipocytes differentiated ex vivo. Stromal cells were isolated from the iWAT of WT and Hepc KO animals and differentiated towards beige adipocytes ex vivo. mRNA levels of thermogenesis genes were analysed by real time PCR after calibration to *Cyclophilin-a*/*Ppia* mRNA. Changes in transcript levels are expressed relative to WT. Error bars represent SEM for n = 3 mice in each group. Statistical significance is indicated by * symbols (*p < 0.05, **p < 0.01).

To further determine the extent to which impaired de novo beige adipogenesis from adipocyte precursors could be responsible for the defect observed in vivo in iWAT of Hepc KO mice, SVF cells were isolated from iWAT and differentiated towards beige adipocytes ex vivo. The treatment was applied for 6 days to ensure full browning of adipocytes. In this setting, SVF progenitors from hepcidin deficient mice failed to undergo normal beige adipocyte thermogenesis, as evidenced by a significant reduction, above two-fold, of *Ucp1*, as well as several other beige-related adipogenesis related mRNAs (Fig. 7). This suggests that hepcidin supports beige adipocyte formation from precursors in a cell intrinsic manner. To further analyse the thermogenic activity of these cells, it would be of value to measure the number and the size of lipid droplets and to quantify lipid accumulation.

The precise mechanism through which hepcidin promotes adipogenic commitment of precursor cells will be an exciting question to address. The possibility that iron loading of precursor cells in the iWAT of Hepc KO mice may alter their adipogenic capacity is of particular interest. Indeed, recent studies have reported the detrimental effect of iron excess on the functionality and differentiation potential of stem cells^36^. In that respect, it was interesting to note the presence of high iron levels in blood vessel walls of AT of Hepc KO mice, since many adipose precursors contributing to beige adipogenesis reside near the vasculature^37,38^.

One potential candidate for future investigation is the multifunctional DNA/RNA binding protein Y-box binding protein 1 (YBX1). Rabiee et al. recently identified YBX1 as a key player in beige adipogenesis^39^. The authors found that YBX1 silencing in differentiating cells had a major impact on the expression of thermogenesis-related genes such as *Pgc1α*, *Elovl3*, *Prdm16*, *Cidea*, and *Ucp1*. Interestingly, *Ybx1* mRNA level was significantly decreased in the iWAT of Hepc KO versus WT mice (Supplemental Fig. 4). Altered regulation of this factor in hepcidin deficiency is particularly interesting in view of the previous report that YBX1 could directly interact with IRP2 in the presence of iron^40^.

In sum, iWAT, but not BAT, of Hepc KO mice exhibited a basal defect in thermogenesis, hallmarked by an altered beige signature with reduced expression of UCP1, the most reliable indicator of thermogenic capacity, and decreased mitochondrial respiration. Upon beta3 adrenergic stimulation, a challenge that predominantly induces transdifferentiation, UCP1 activation in the iWAT of Hepc KO mice was restored. In contrast, the thermogenic activity of the iWAT was persistently deficient in Hepc KO animals exposed to cold, a condition that involves de novo adipogenesis. This suggests that the WAT browning defect in hepcidin deficiency results from impaired de novo adipogenesis, possibly due to iron overload of precursor cells and subsequent alteration of their differentiation properties (Supplementary Fig. 5). Recent characterization of body fat compartments at single-cell resolution has enabled a broader and deeper understanding of the cell sub-populations that constitute adipose tissue, in particular adipose stem cells, offering new insights into the regulatory mechanisms within adipose tissue^41,42^. Such single cell approaches should provide valuable insights into how hepcidin defficiency may affect the different cell types that reside in adipose tissue.

## Methods

### Animals

Ethic statement. Mice were cared for in accordance with the principles and guidelines established by the European convention for the protection of laboratory animals (Directive 2010/63/EU of the European Parliament and of the Council). Animal studies received approval from the Regional Ethics Committee for Animal Experimentation of Université Paris Cité. Reporting of animal data in this study followed the recommandations set out in the ARRIVE guidelines.

Mice were maintained in a specific pathogen-free animal facility on a 12 h light/dark cycle. Animals were given free access to tap water and a standard laboratory mouse chow diet (AO3, iron content 280 mg/kg, UAR, France). Age-matched wild-type (WT) and homozygote knock-out (Hepc KO) male mice (35–45-weeks of age) on a C57BL/6 background were used in this study^25^.

For cold exposure, mice were housed individually at 4 °C for 48h, with free access to tap water and food.

For b3-adrenergic receptor agonist treatment, mice were injected intraperitoneally with 1 mg of CL316,243 (Merck, C5976) per kg of body weight daily during 5 days, and were sacrificed 5 h after the last injection. Mice injected with PBS served as reference.

### Iron concentration

Plasma and tissue iron content were measured on an Olympus AU400 automat using a colorimetric method. Tissue iron was determined after acid digestion of the samples, and iron levels are presented relative to wet weight.

### Plasma cytokines quantification

Mice plasma were analysed with the V-PLEX Proinflammatory Panel1 (mouse) kit from Meso Scale Discovery (K15048D-1) according to the manufacturer’s recommendations.

### SVF and adipocytes preparation from mouse inguinal adipose tissue

Subcutaneous inguinal adipose tissues were minced with scissors after removal of the lymph node. Tissue pieces were digested for 1 h at 37 °C in DMEM containing 2 mg/mL collagenase Type I (Gibco, 17100-017) with constant agitation (150 rpm) in a shaker. Digestion was stopped by adding one volume of DMEM supplemented with 20% fœtal calf serum. The cell suspension was strained through a 70 µm nylon mesh and centrifuged at 250*g* for 10 min at 20 °C to separate SVF from mature adipocytes. Adipocytes floating cells were harvested and SVF was recovered in the pellet. The two fractions were frozen in lysis buffer (50 mM Tris, pH 7.4, 1% Triton X-100, 150 mM NaCl, 10% glycerol, 50 mM NaF, 5 mM sodium pyrophosphate, 1 mM Na_3_VO_4_, 25 mM sodium-β-glycerophosphate, 1 mM DTT) containing protease and phosphatase inhibitors.

For in vitro adipocytes differentiation, SVF were seeded in high glucose DMEM (Gibco, 31966-021) complemented with 10% fœtal calf serum, penicillin and streptomycin (100 U/mL each) and kept in culture for 1 week. Cells were then harvested and placed in six-well plates in high glucose DMEM supplemented with 5 mg/mL Insulin (Novo Nordisk, Actrapid), 0.5 mM 3-Isobutyl-1-methylxanthine [IBMX] (Merck, I5879), 100nM 3,3′,5-Triiodo-l-thyronine [T3] solution (Merck, T-074), 1 µM dexamethasone (Merck, D4902), 1 µM Rosiglitazone (Merck, R2408). After 4 days in culture, cells were maintained for 2 days in high glucose DMEM supplemented with 5 mg/mL Insulin, 100 nM T3 solution and 1 µM Rosiglitazone.

Effects of iron loading on adipocytes were studied by addition of Fe-NTA (100 µM FeCl_3_-400 µM NTA; Merck F1513 and N0128 respectively) in fresh culture media for 18 h.

### Immunochemistry

Tissues were fixed in 4% formaldehyde and embedded in paraffin. Immunostaining was performed using 4 µm thick tissue sections. For iron detection, slides were stained with Perls’ Prussian blue (together with nuclear fast red as counterstain) using standard procedures. For αSMA immune-staining, endogenous peroxidases were neutralized in 3% H_2_O_2_ for 20 min. Tissue sections were permeabilised for 20 min in PBS containing 0.5% Triton X, then blocked for 30 min at room temperature in PBS containing 3% BSA, 0.1% Triton X-100, and 10% normal goat serum. Sections were incubated at 4 °C over night with primary antibody against αSMA (A5228 from Merck, diluted 1:200 in PBS + 3% BSA, 0.1% Triton X-100, 1% normal goat serum). Immune complexes were detected using a HRP-conjugated secondary antibodies and the ImmPACT NovaRED Peroxidase Substrate Kit (Vector Laboratories, Burlingame, CA, USA) according to the manufacturer’s instructions. Counterstaining was performed with Nuclear Fast Red (Vector Laboratories). Images were acquired using the Lamina Slide scanner (Akoya Perkin Elmer) and analysed with Case Viewer software (3DHistech).

### Reverse transcription and real-time PCR

RNA extraction, reverse transcription, and real-time PCR were performed as previously described^43^. Briefly, total RNA was isolated with TriReagent (Molecular Research Center, Cincinnati, OH, USA), and reverse transcribed using the High Capacity cDNA Starter Kit, (4368813 from Applied Biosystems) according to the manufacturer’s instructions. Real-time PCR was performed in a LightCycler 480 Instrument II (Roche) using the SYBR Green PCR mix (4368813 from Roche) in accordance with the MIQE guidelines^44^. Relative mRNA levels were determined by the second derivative maximum method with the LightCycler 480 analysis software. All samples were normalised to the threshold cycle value for *Cyclophilin-a* (*Ppia*). Primer sequences used in this study are provided in Table 1.

**Table1:** Primer sequences used in this study.

Gene	Forward	Reverse
*Irp1/Aco1*	AATTCGAGGACTCAAGATACGG	TCTTCACCAGAAACTCATCACAG
*Cidea*	TGCTCTTCTGTATCGCCCAGT	GCCGTGTTAAGGAATCTGCTG
*Cox7a*	CAGCGTCATGGTCAGTCTGT	AGAAAACCGTGTGGCAGAGA
*Cox8b*	GAACCATGAAGCCAACGACT	GCGAAGTTCACAGTGGTTCC
*Cpt1b*	GCTGCCGTGGGACATTC	CTTGGCTACTTGGTACGAGTTCTC
*Cyclophilin-a*/*Ppia*	ATGGCACTGGCGGCAGGTCC	TTGCCATTCCTGGACCCAAA
*Dio2*	CTGCGCTGTGTCTGGAAC	GGAGCATCTTCACCCAGTTT
*Elovl3*	TCCGCGTTCTCATGTAGGTCT	GGACCTGATGCAACCCTATGA
*Ftl1*	GGGCCTCCTACACCTACCTC	CTCCTGGGTTTTACCCCATT
*Irp2/Ireb2*	CCCGTGTTGTTCTTCAAGATTT	TCTTAGGATCACCTCCAAGAGTTT
*Pgc1α*/*Ppargc1a*	ATACCGCAAAGAGCACGAGAAG	CTCAAGAGCAGCGAAAGCGTCACAG
*Pparα*/*Ppara*	CCCTGTTTGTGGCTGCTATAATTT	GGGAAGAGGAAGGTGTCATCTG
*Prdm16*	GCCATTCATATGCGAGGTCT	CCAGGCGTGTAATGGTTCTT
*Tfr1*/*Tfrc*	CGTGATCAACATTTTGTTAAGATTCA	CCACATAACCCCCAGGATTCT
*Ucp1*	ACTGCCACACCTCCAGTCATT	CTTTGCCTCACTCAGGATTGG

### Nuclear and mitochondrial DNA quantification

Brown and inguinal adipose tissues were incubated at 56 °C for 24 h in lysis buffer (100 mM Tris–HCl, 5 mM EDTA, 200 mM NaCl, 0.2% SDS, 0.1 mg/mL proteinase K), separated by isopropanol precipitation, washed with 70% ethanol, and stored in pure water. The expression of nuclear (HK2) and mitochondrial (mt 16S RNA) genes was analysed by real time PCR using 0.5 ng of gDNA and the following primers HK2-F: GCCAGCCTCTCCTGATTTTAGTGT; HK-2 R: GGGAACACAAAAGACCTCTTCTGG; mt16S RNA F: CCGCAAGGGAAAGATGAAAGAC; mt16S RNA R: TCGTTTGGTTTCGGGGTTTC.

### Western Blot (WB) analysis

Total lysates from inguinal white adipose tissue and brown adipose tissue were homogenised in lysis buffer (50 mM Tris, pH 7.4, 1% Triton X-100, 150 mM NaCl, 10% glycerol, 50 mM NaF, 5 mM sodium pyrophosphate, 1 mM Na_3_VO_4_, 25 mM sodium-β-glycerophosphate, 1 mM DTT) supplemented with protease and phosphatase inhibitors. Lysates were centrifuged at 7500 rpm three times, to eliminate lipids, and sonicated. Cytosolic extracts were obtained from total lysates centrifuged at 41,000 rpm for 1 h. Samples were analysed by SDS-PAGE and transferred onto a nitrocellulose membrane in Tris/glycine buffer. Blocking of the membrane was performed in 10 mM Tris-buffered saline (pH 7.4) containing 0.05% NP40 (TBSNP) and 5% (w/v) non-fat milk powder. All primary antibodies were incubated with the membrane overnight at 4 °C on a rocking platform. Membranes were washed in TBS + 0.05% Tween20 and probed with a HRP-conjugated secondary antibody in TBSNP + 5% (w/v) non-fat milk for 1 h at room temperature. Chemiluminescence was detected with Clarity Western ECL Substrate (Bio-Rad). Proteins were visualised with Image Quant Las4000 mini (GE Healthcare). Densitometry of the immunoblots was performed using ImageJ software. Antibodies used in the study are listed in Table 2. Original blots are presented in the Supplementary Information.

**Table 2:** List of the antibodies used in this study.

Protein	Supplier	Reference
ACO1	Kind gift by Bruno Galy	
αTubulin	Merck	SAB4500087
βActin	Merck	A2066
DMT1	Kind gift by François Canonne Hergaux	
FBXL5	Invitrogen	PA5-42296
l-Ferritin	Merck	SAB2500431
Ferroportin	Alpha Diagnostics Int	MTP11A
IREB2	Kind gift by Bruno Galy	
PGC1α	Abcam	Ab54481
TFR1	Abcam	AB84036
UCP1	Kind gift by Daniel Riquier	
VDAC1	Abcam	Ab14734

### Oxygen consumption

Brown and adipocyte fresh tissues were collected, weighed, cut in small pieces and resuspended in respiration buffer (50 mM KCl, 20 mM sucrose, 5 mM TES, 2.5 mM MgCl_2_, 0.5 mM EGTA, 1% BSA fatty acid free, 10 mM KPi) as follows: 5 µL or 20 µL of buffer per mg of, respectively, iWAT or BAT. Samples were homogenised by PBI-shredder in a schredder tube with a metal lysis disk during 10 s at position 1. The oxygen consumption was measured at 25 °C in the 2 mL respiratory chamber of an Oroboros O2k oxygraph (OROBOROS INSTRUMENTS, Austria). The homogenated tissue was transferred in a chamber of O2k, and the volume was adjusted to 2 mL before closing the stopper. Mitochondria respiration was activated by adding 7 mM succinate (Merck, S-2370) and 1.56 mM ADP (Merck, A-2754). Maximum respiratory rate was determined by adding increasing amounts of CCCP (Merck, C-2759). Respiratory chain was inhibited after addition of 1 mM of potassium cyanide. The coupling status was quantified by the ratio between the basal rate of oxygen consumption in presence of succinate alone and either the rate after addition of ADP or the maximum respiratory rate.

### Statistical analysis

Data are expressed as the mean ± S.E.M. Statistical analysis, unless otherwise stated, was performed using Student t test (unpaired, 2 tailed) with GraphPad Prism software v9 (GraphPad Software). p values less than 0.05 were considered statistically significant, *p < 0.05; **p < 0.01, ***p < 0.001 and ****p < 0.0001).

### Supplementary Information

Supplementary Figures.

## Data Availability

The datasets used and/or analysed during the current study are available from the corresponding author on reasonable request.
