# Supplementary material for: Hepcidin deficiency in mice impairs white adipose tissue browning possibly due to a defect in de novo adipogenesis
Source: Sci Rep. 2023 Aug 7;13:12794. doi: 10.1038/s41598-023-39305-0 (PMC10406828; doi:10.1038/s41598-023-39305-0)

Hepcidin deficiency in mice impairs white adipose tissue browning due to a defect in de novo adipogenesis

Jean-Christophe Deschemin[1,2], Celine Ransy[1,2], Frederic Bouillaud[1,2], Soonkyu Chung[3], Bruno Galy[4], Carole Peyssonnaux[1,2] and Sophie Vaulont[1,2] *

Affiliations:

[1]Université Paris Cité, CNRS, INSERM, Institut Cochin, F-75014 Paris, France

[2]Laboratory of Excellence GR-Ex, Paris, France.

[3]Department of Nutrition and Health Sciences and Department of Biochemistry at the University of Nebraska, Lincoln Nebraska 68583, USA

[4]German Cancer Research Center, "Division of Virus-Associated Carcinogenesis", Im Neuenheimer Feld 280, 69120 Heidelberg, Germany.

*Corresponding author. Email: sophie.vaulont@inserm.fr

Supplemental Figures

Supplemental Fig.1: Expression of iron metabolism genes in iWAT and BAT of WT and Hepc KO mice

Real time PCR analysis of *Irp1* (left) and *Irp2* (right) mRNA levels in iWAT (top) and BAT (bottom) from WT and Hepc KO mice after calibration to *Cyclophilin-a/Ppia* mRNA. Expression changes are presented relative to WT.

Error bars represent SEM for n=3 mice in each group. Statistical significance is indicated by * symbols (*$p < 0.05$).

Supplemental Fig.2: Expression of iron and thermogenic genes in isolated adipocytes from iWAT of WT and Hepc KO mice

Real time PCR analysis of *Tfr1*, *Pgc1*α, and *Ucp1* mRNA levels in primary cultures of mature adipocytes treated with iron (Fe-NTA) for 18h. Changes are expressed relative to non treated cells after calibration to *Cyclophilin-a/Ppia* mRNA.

Error bars represent SEM for n=3 mice in each group. Statistical significance is indicated by * symbols (*$p < 0.05$, ***$p < 0.0001$).

Supplemental Fig.3: Analysis of the inflammatory status in the iWAT and the plasma of WT and Hepc KO mice

(A) Real time PCR analysis of *Il6*, *Tnf*α, and *Il1*β mRNA levels in the iWAT of Hepc KO mice. Changes are expressed relative to WT mice after calibration to *Cyclophilin-a/Ppia* mRNA.

(B) IL6, TNFα and IL1β cytokine levels in plasma of WT versus Hepc KO mice.

Error bars represent SEM for n=5 mice in each group. ns is for not significant.

Supplemental Fig.4: Expression of *Ybx1* levels in iWAT of WT and Hepc KO mice

Real time PCR analysis of *Ybx1* mRNA levels in iWAT of WT and Hepc KO mice. Changes are expressed relative to WT after calibration to *Cyclophilin-a/Ppia* mRNA.

Error bars represent SEM for n=3 mice in each group. Statistical significance is indicated by * symbols (*$p < 0.05$, **$p < 0.001$).

Supplemental Fig.5: Schematic representation of WAT browning alterations in Hepc KO mice

SUPPLEMENTAL FIGURE 1

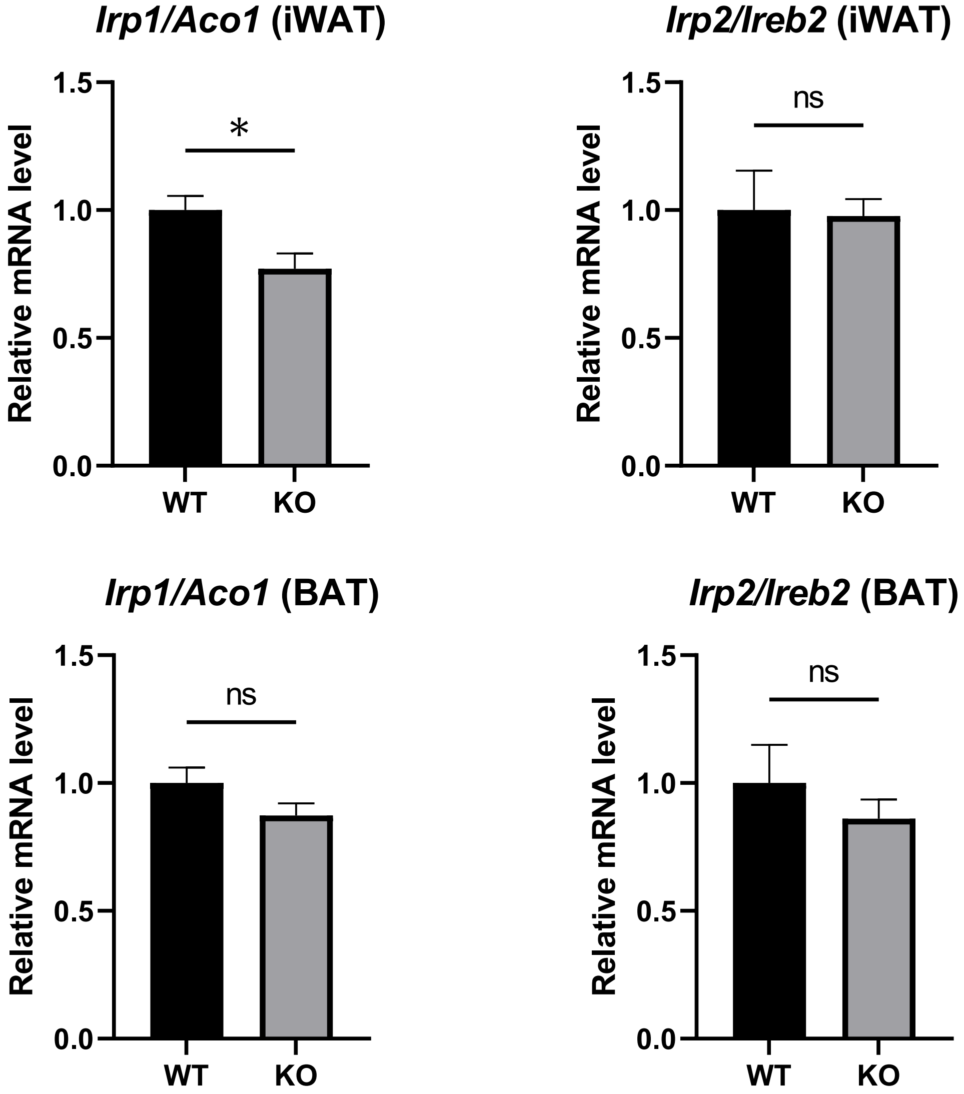

SUPPLEMENTAL FIGURE 2

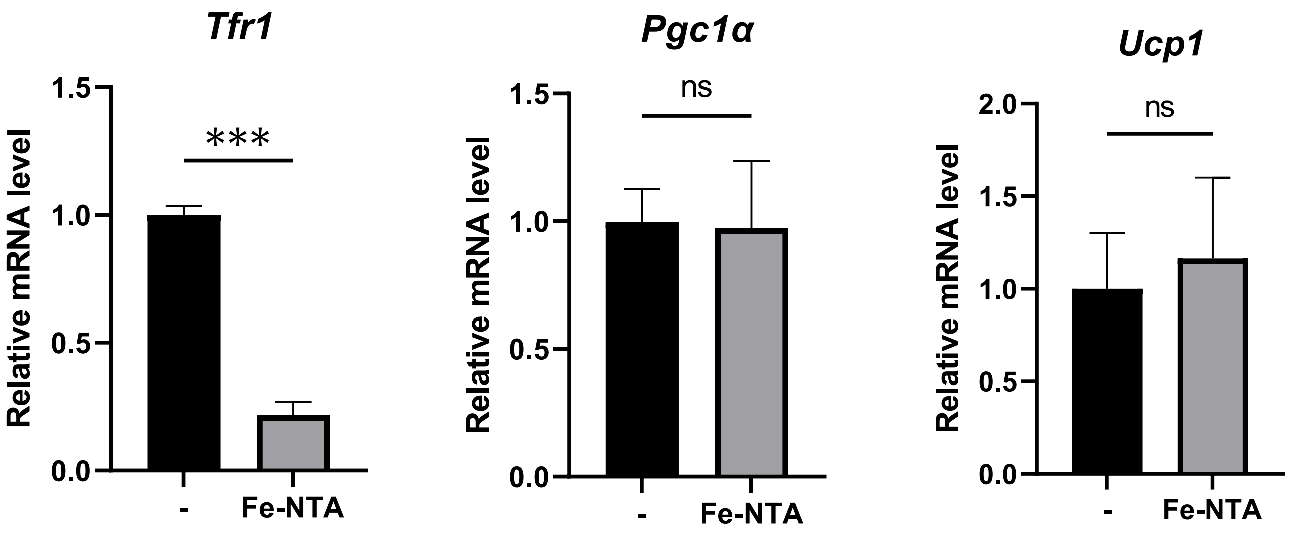

SUPPLEMENTAL FIGURE 3

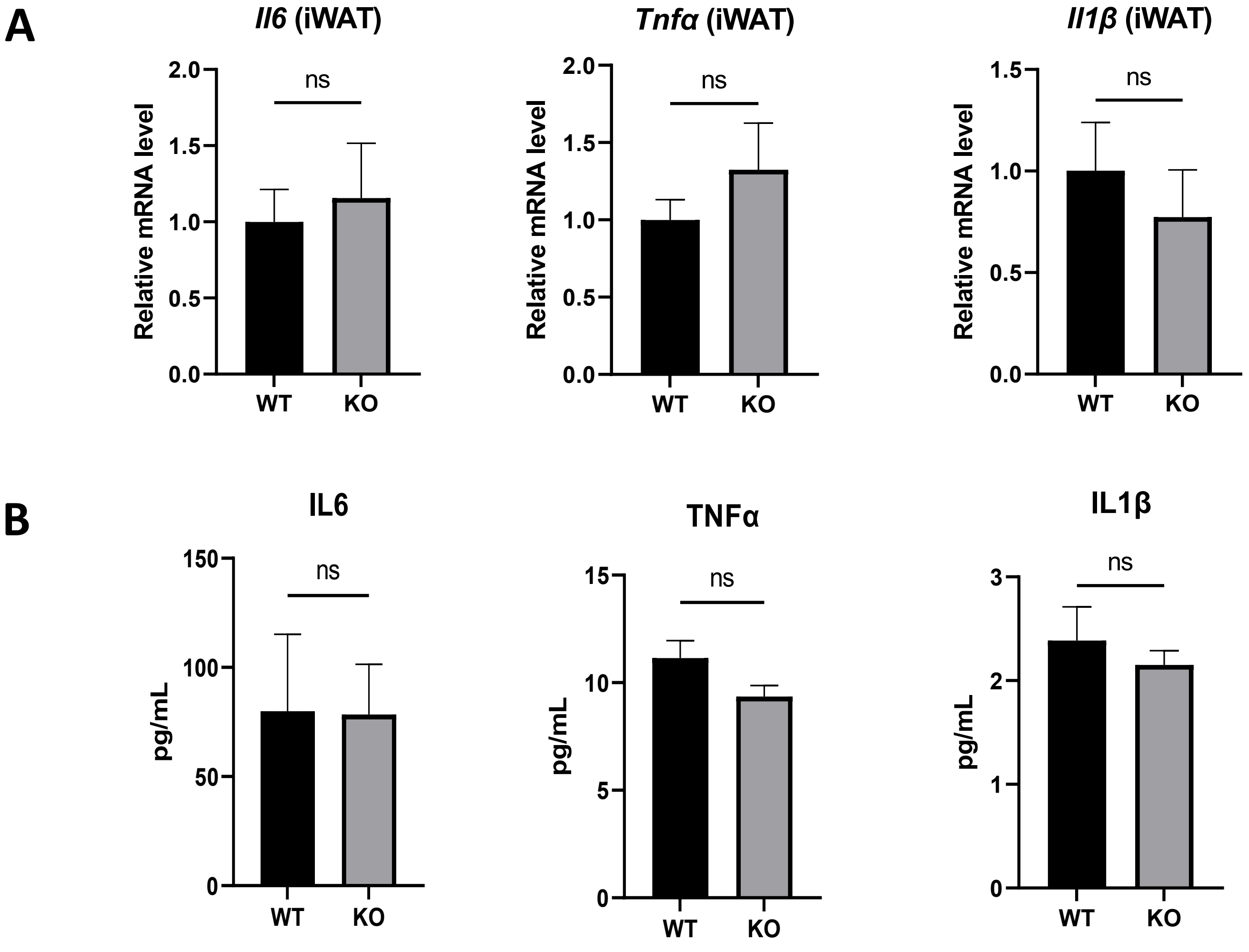

SUPPLEMENTAL FIGURE 4

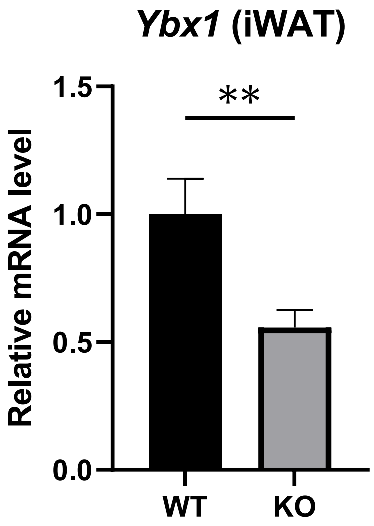

SUPPLEMENTAL FIGURE 5

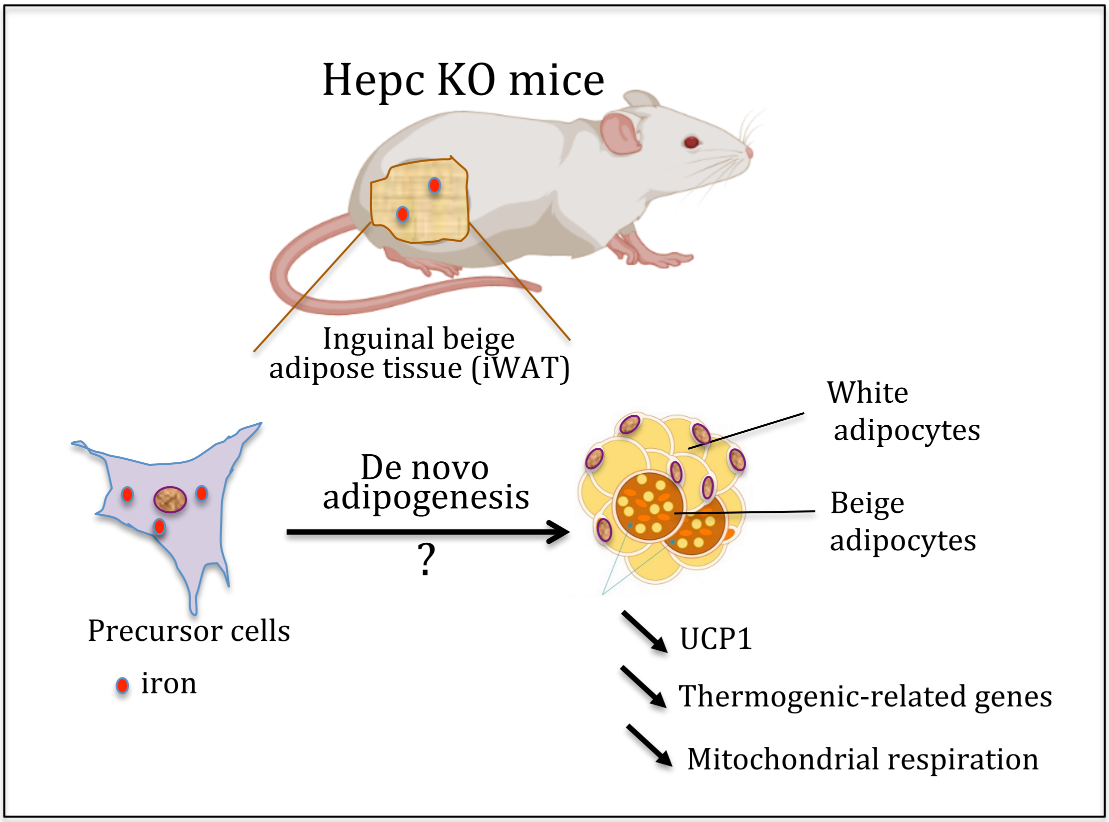

ORIGINAL BLOTS USED FOR Fig 1 TO Fig 6

B

Fig.1

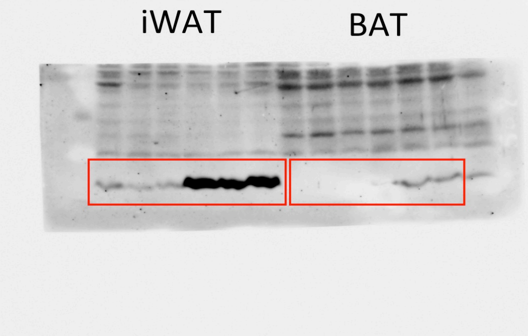

L-Ferritin

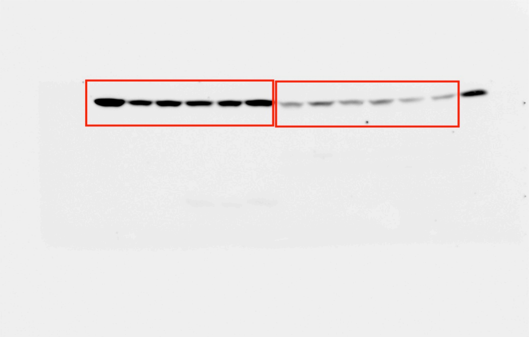

β-actin

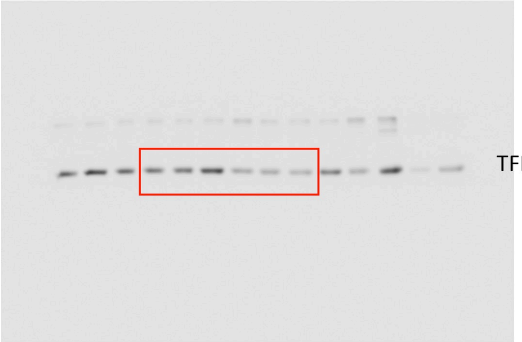

TFR1

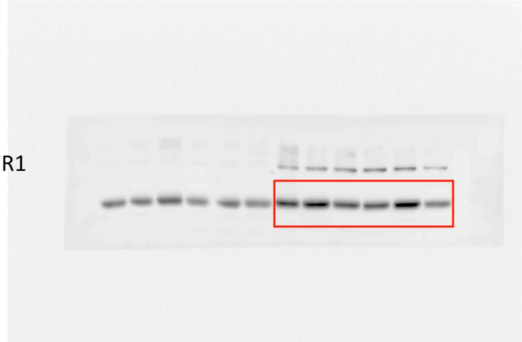

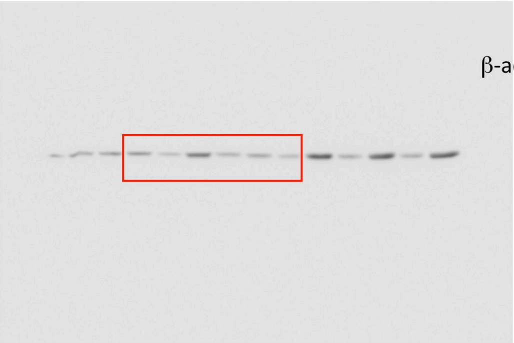

β-actin

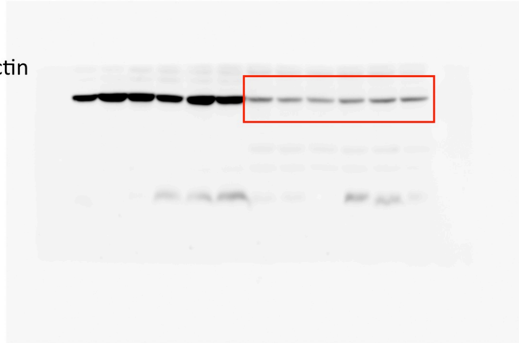

Fig.1

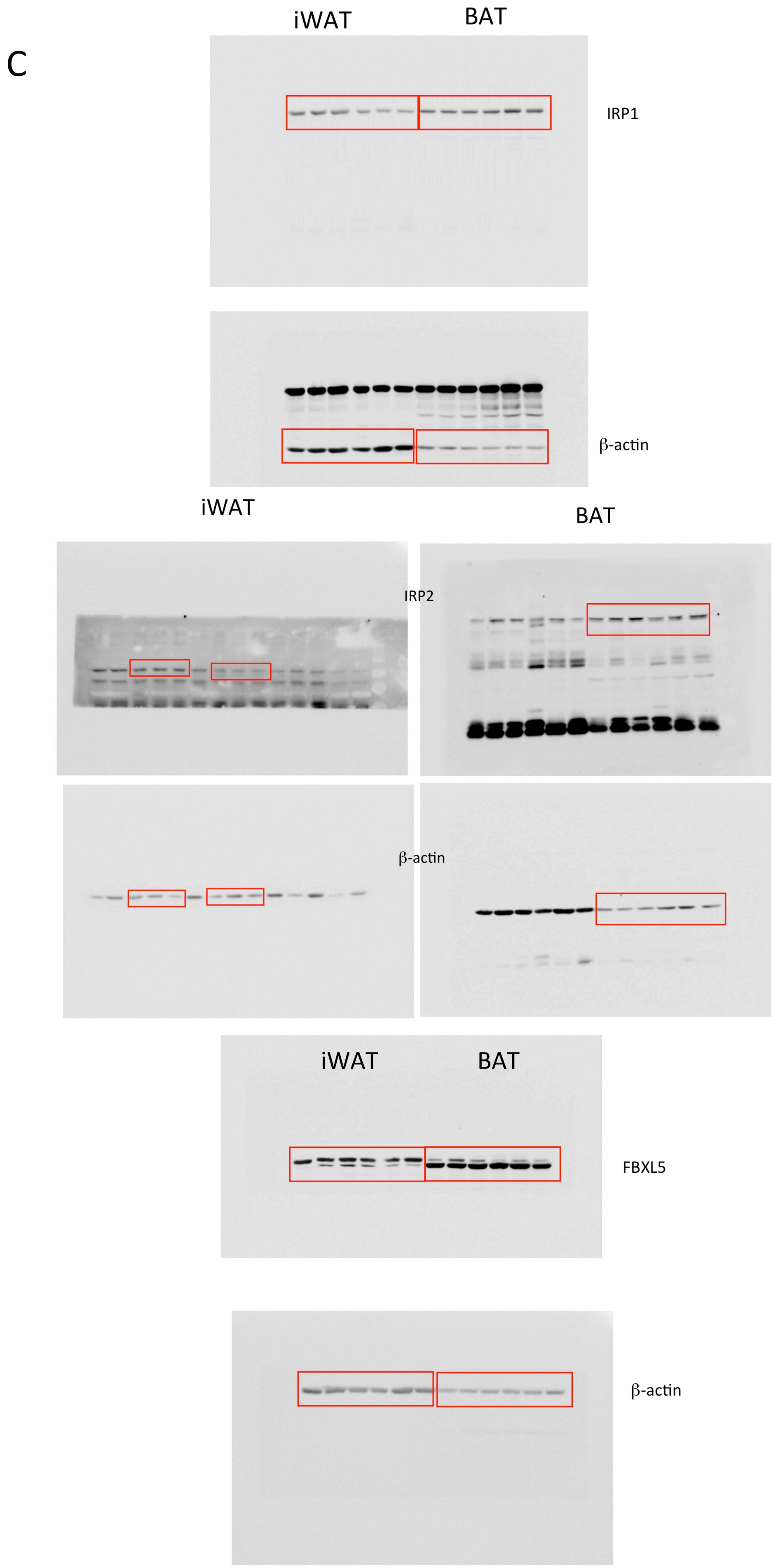
C
iWAT
BAT
IRP1
β-actin
iWAT
BAT
IRP2
β-actin
iWAT
BAT
FBXL5
β-actin

Fig.1

D

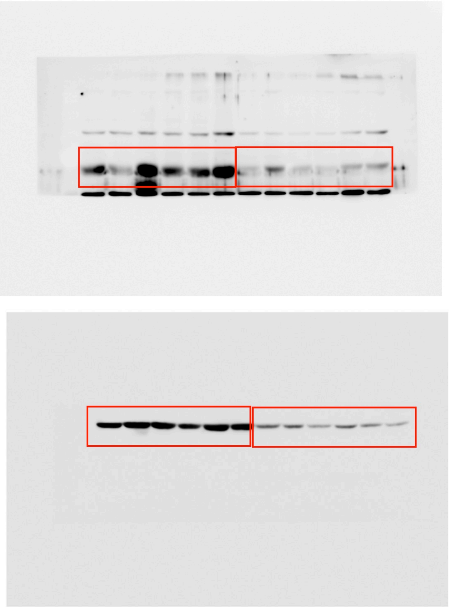

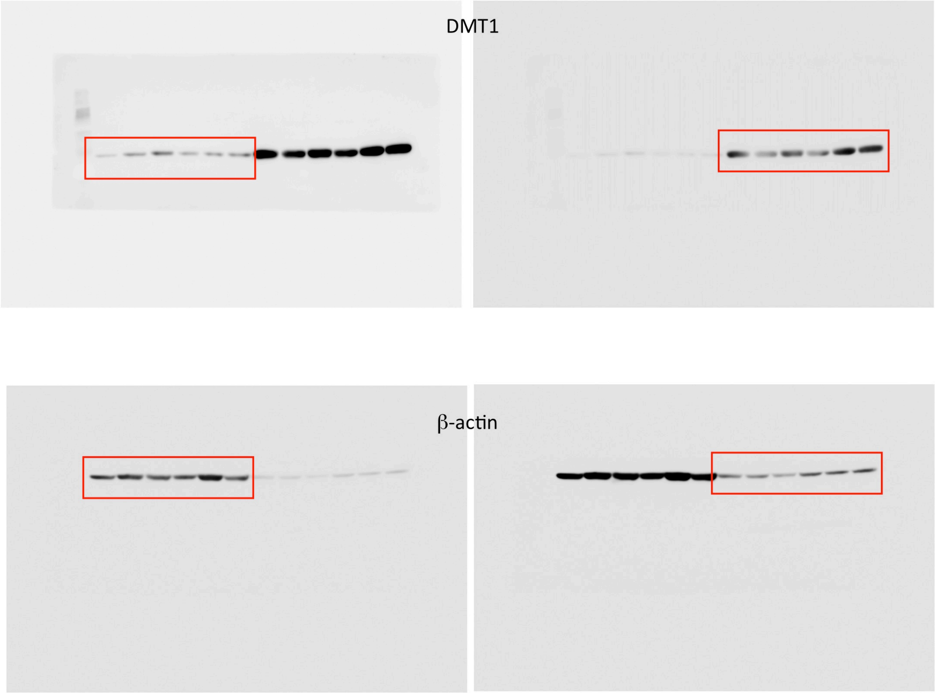

Fig.2

A

iWAT

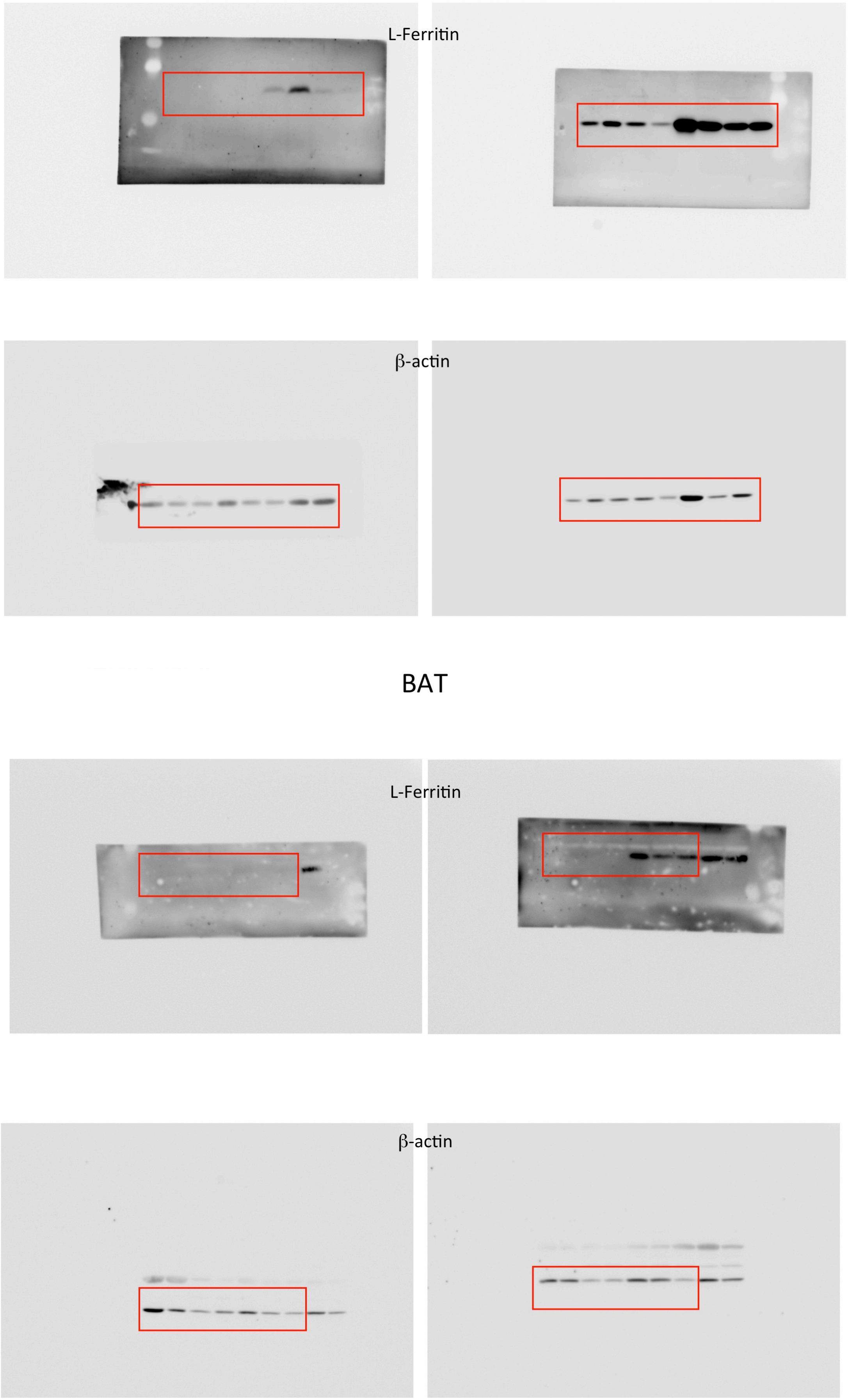

Fig.3

A

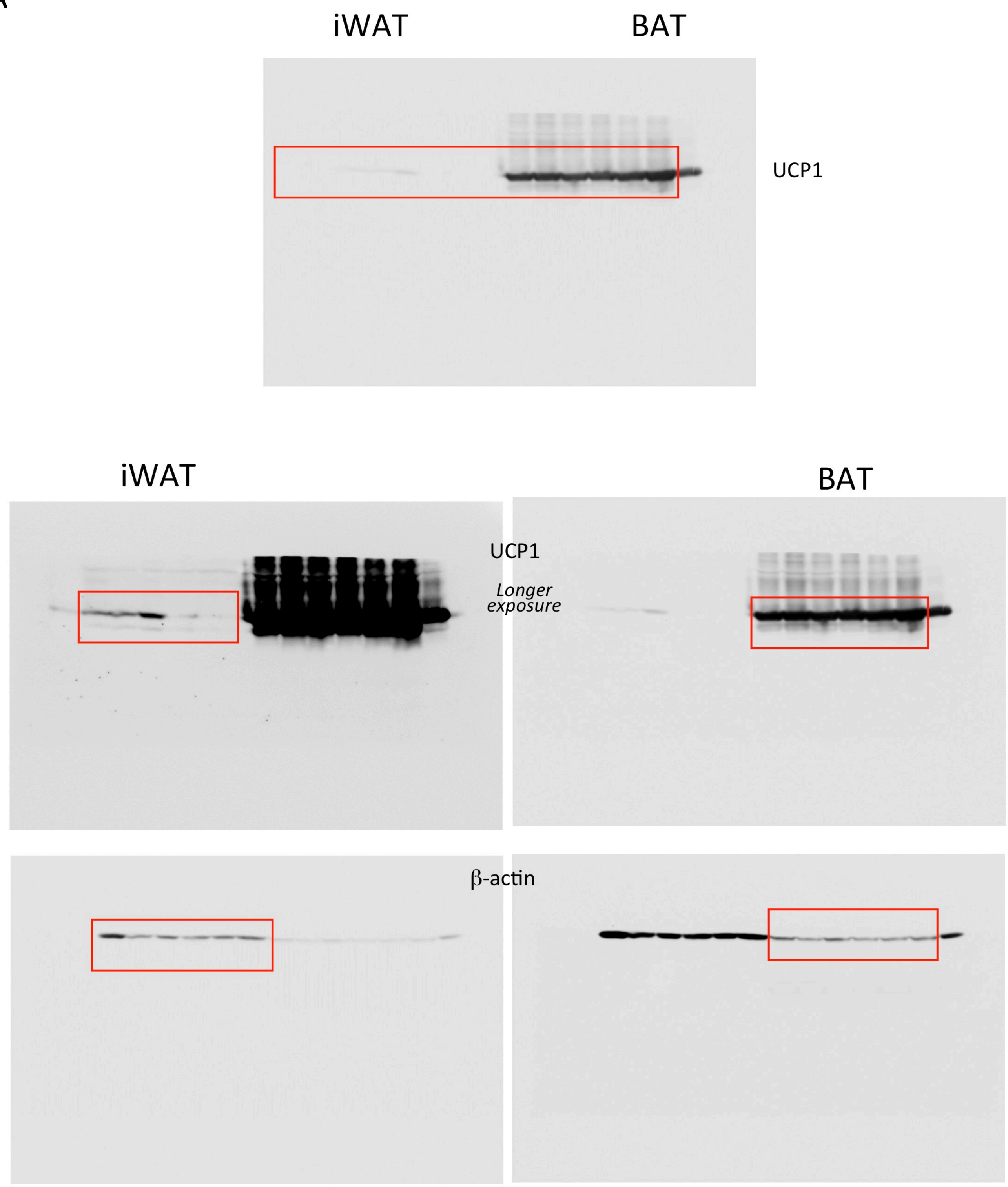

Fig.3

D

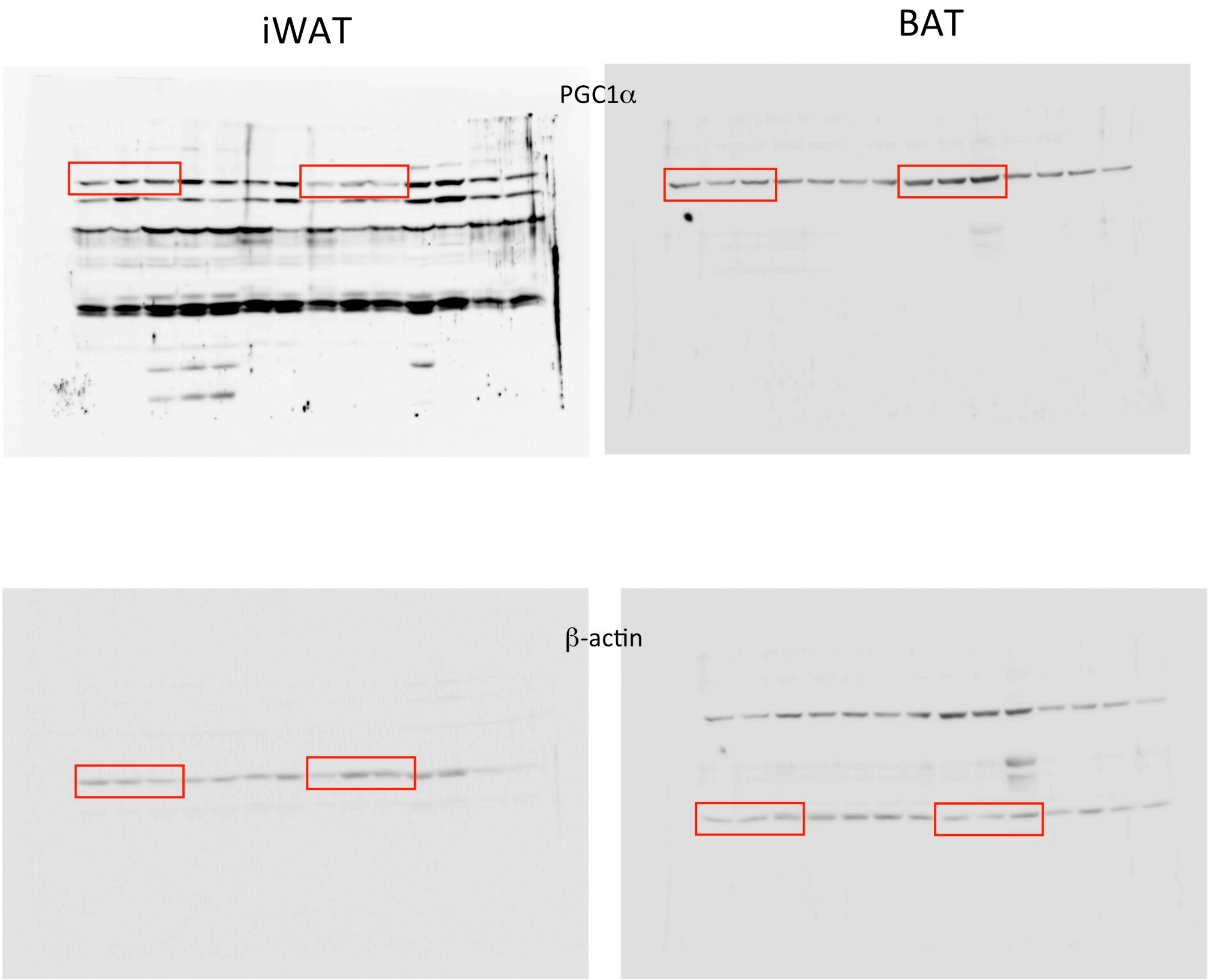

Fig.4

B

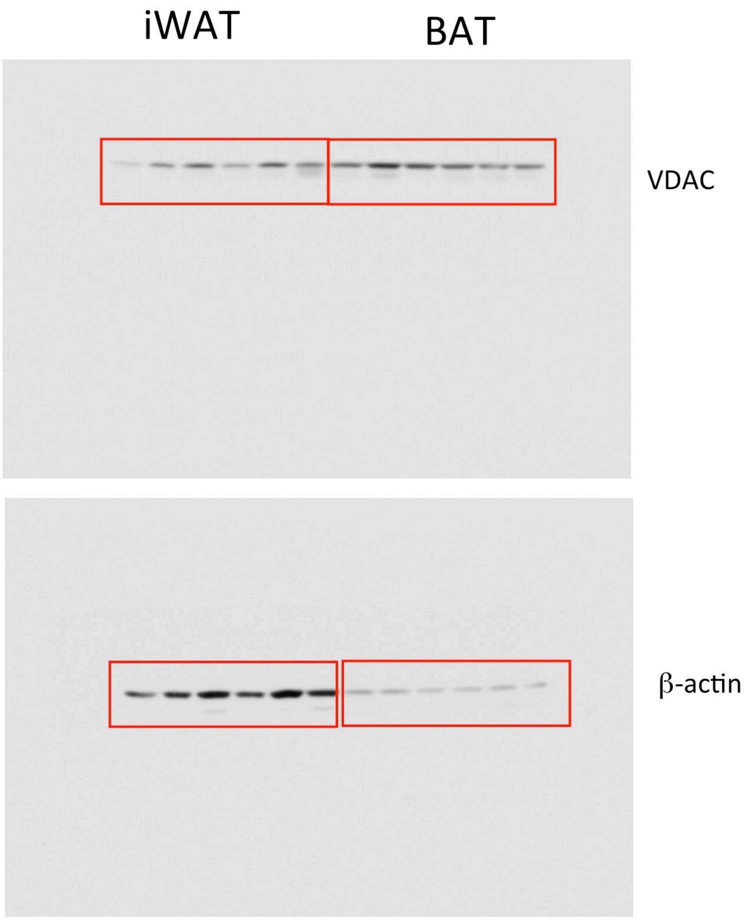

Fig.5

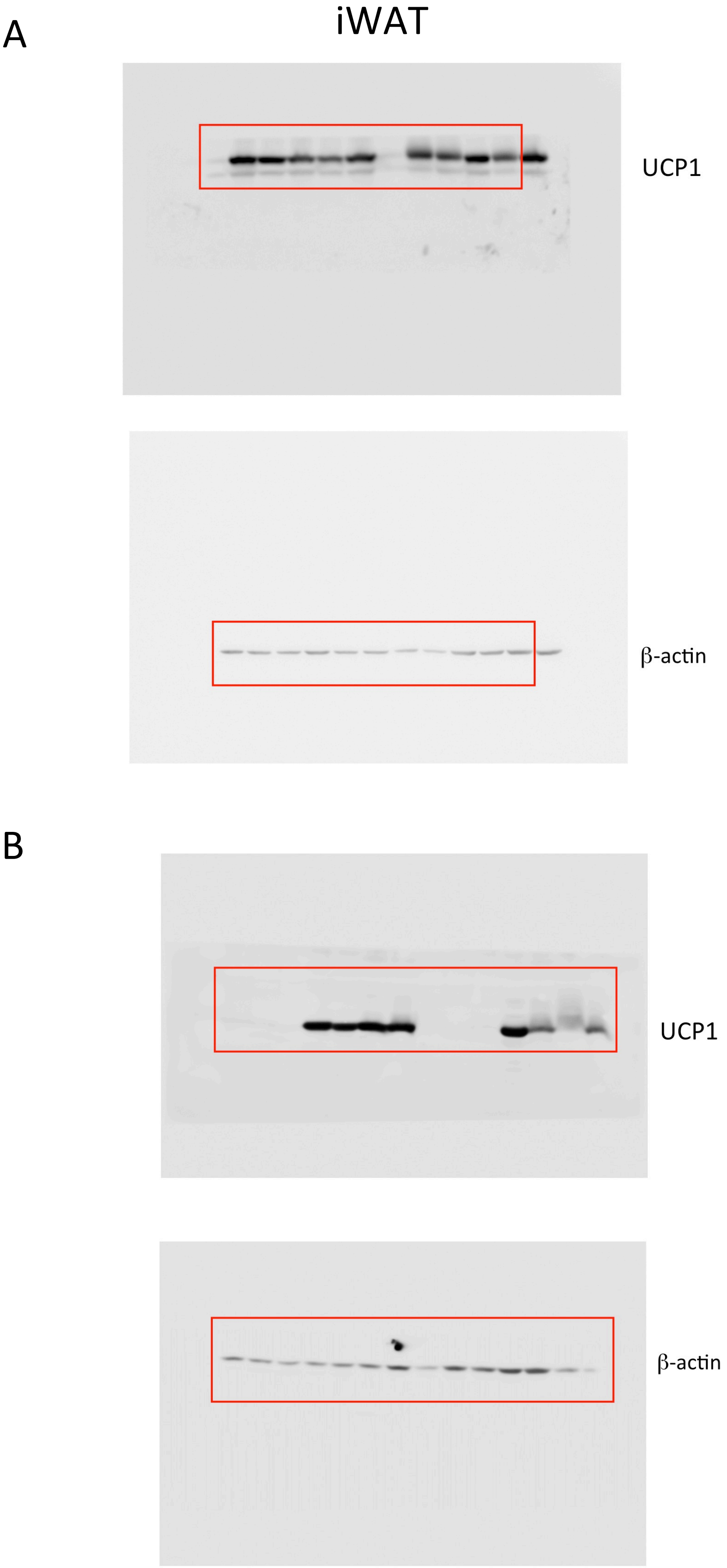

Fig.6

A iWAT

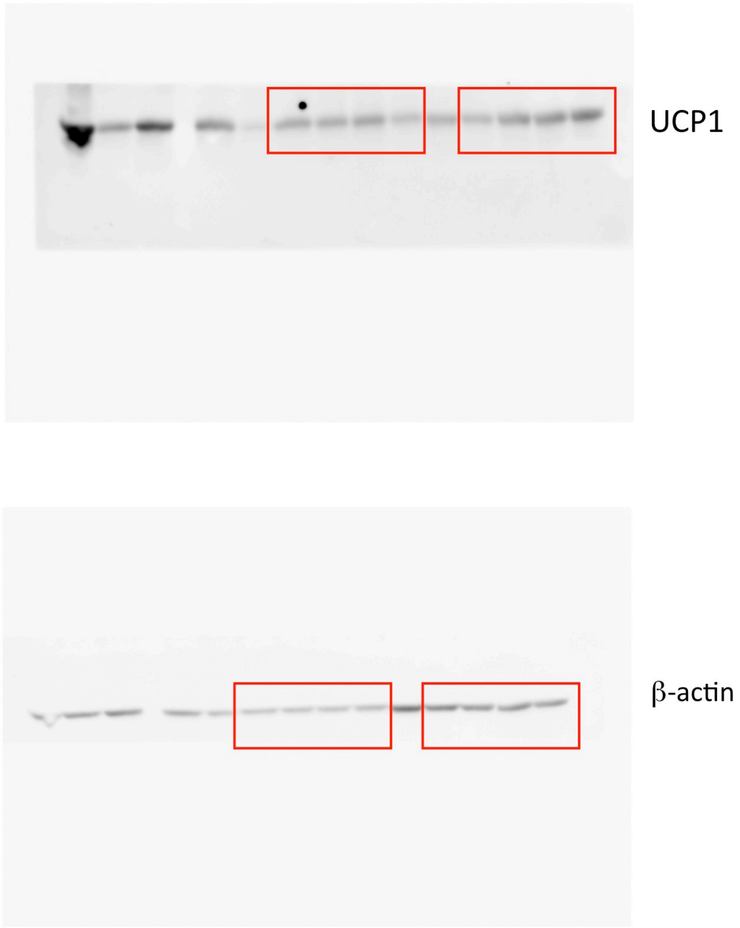

Supplement: Supplementary file 1 — Supplementary Figures. [file 41598_2023_39305_MOESM1_ESM.pdf]
